# High-Throughput Sequencing Analysis of Post-Liver Transplantation HCV E2 Glycoprotein Evolution in the Presence and Absence of Neutralizing Monoclonal Antibody

**DOI:** 10.1371/journal.pone.0100325

**Published:** 2014-06-23

**Authors:** Gregory J. Babcock, Sowmya Iyer, Heidi L. Smith, Yang Wang, Kirk Rowley, Donna M. Ambrosino, Phillip D. Zamore, Brian G. Pierce, Deborah C. Molrine, Zhiping Weng

**Affiliations:** 1 MassBiologics, University of Massachusetts Medical School, Boston, Massachusetts, United States of America; 2 Program in Bioinformatics and Integrative Biology, University of Massachusetts Medical School, Worcester, Massachusetts, United States of America; 3 Department of Biochemistry and Molecular Pharmacology, University of Massachusetts Medical School, Worcester, Massachusetts, United States of America; 4 Bioinformatics Program, Boston University, Boston, Massachusetts, United States of America; SRI International, United States of America

## Abstract

Chronic hepatitis C virus (HCV) infection is the most common cause of end-stage liver disease, often leading to liver transplantation, in which case circulating virions typically infect the transplanted liver within hours and viral concentrations can quickly exceed pre-transplant levels. MBL-HCV1 is a fully human monoclonal antibody recognizing a linear epitope of the HCV E2 envelope glycoprotein (amino acids 412–423). The ability of MBL-HCV1 to prevent HCV recurrence after liver transplantation was investigated in a phase 2 randomized clinical trial evaluating six MBL-HCV1-treated subjects and five placebo-treated subjects. MBL-HCV1 treatment significantly delayed time to viral rebound compared with placebo treatment. Here we report results from high-throughput sequencing on the serum of each of the eleven enrolled subjects prior to liver transplantation and after viral rebound. We further sequenced the sera of the MBL-HCV1-treated subjects at various interim time points to study the evolution of antibody-resistant viral variants. We detected mutations at one of two positions within the antibody epitope—mutations of N at position 415 to D, K or S, or mutation of N at position 417 to S. It has been previously reported that N415 is not glycosylated in the wild-type E2 protein, but N417S can lead to glycosylation at position 415. Thus N415 is a key position for antibody recognition and the only routes we identified for viral escape, within the constraints of HCV fitness in vivo, involve mutating or glycosylating this position. Evaluation of mutations along the entire E1 and E2 proteins revealed additional positions that changed moderately before and after MBL-HCV1 treatment for subsets of the six subjects, yet underscored the relative importance of position 415 in MBL-HCV1 resistance.

## Introduction

Chronic hepatitis C virus (HCV) infection is the most common cause of end-stage liver disease leading to liver transplantation in the United States [1]. Unfortunately, recurrence of hepatitis C infection post-transplantation is nearly universal. While serum HCV RNA levels initially decline with removal of the infected liver, circulating virions infect the donor liver within hours and viral concentrations increase rapidly in most patients, often exceeding pre-transplant levels [2], [3]. Recurrent HCV disease is often more aggressive in the setting of post-transplant immunosuppression, with accelerated cirrhosis, increased risk of graft failure, and death [4], [5].

MBL-HCV1 is a novel fully human IgG1/kappa monoclonal antibody (MAb) isolated from mice expressing human antibody genes (Medarex, Inc., a wholly owned subsidiary of Bristol-Myers Squibb). MBL-HCV1 binds a highly-conserved linear epitope of the HCV E2 envelope glycoprotein (amino acids 412–423) and neutralizes a broad range of genotypes in vitro [6]. MBL-HCV1 is capable of preventing HCV infection in a chimpanzee model of acute HCV [7]. Treatment of chronically-infected chimpanzees with a single dose of MBL-HCV1 led to suppression of viral load in a subset of animals for up to 14 days, with viral rebound coinciding with the emergence of antibody-resistant virus. Alterations at amino acid positions 415 (N415K and N415D) and 417 (N417S) within the MBL-HCV1 epitope dominated the viral population in chimpanzees post-treatment [7].

The ability of MBL-HCV1 to prevent HCV recurrence after liver transplantation is being investigated in clinical trials as current treatment options are limited. In a phase 2 randomized, placebo-controlled trial in this target population, treatment with MBL-HCV1 significantly delayed median time to viral rebound compared to placebo treatment [8]. The strong selective pressure of this neutralizing antibody resulted in the emergence of MBL-HCV1 resistance-associated variants (RAVs), as determined by conventional cloning and Sanger sequencing methods in all subjects receiving MAb monotherapy. The time to emergence of detectable RAVs varied from 6 to 42 days and was associated with a rebound in circulating viral titer.

In this article, we use high-throughput next generation sequencing to investigate the presence of resistance mutations to MAb pre-transplant and examine the post-transplant evolution of HCV variants in the presence and absence of MBL-HCV1 antibody (SRA study accession number SRP037575).

## Results

### Analysis of HCV E1/E2 Variants at Time of Viral Rebound

Eleven enrolled subjects underwent liver transplantation in a phase 2 clinical study (Table 1) [8]. Six subjects were randomized to receive MBL-HCV1 (subjects A–F) and five subjects were randomized to placebo (subjects G–K). To assess viral RNA sequences found in serum samples obtained during the clinical study, a high-throughput sequencing methodology was developed. We initially applied high-throughput next generation sequencing to samples obtained following 2 log_10_ viral rebound in MBL-HCV1-treated subjects. For placebo subjects, samples obtained 7–21 days post-transplantation were also sequenced. The frequencies of amino acid alterations at the positions associated with resistance to MBL-HCV1 neutralization were analyzed (**Table 1**).

**Table 1:** Subject characteristics and resistance-associated variants at viral rebound.

Subject	Treatment	Baseline IU/ml (log_10_)	Study day sequenced post-viral rebound	Estimated number of input genomes	Position 415 amino acid (%)	Position 417 amino acid (%)
**A**	MBL-HCV1	6.7	14	2,664,824	**D** (78.5)	N (78.8)
					N (20.8)	**S** (20.5)
**B**	MBL-HCV1	6.1	14	27,214	**K** (97.1)	N (99.0)
					N (1.7)	
**C**	MBL-HCV1	7.2	14	32,037	**D** (56.0)	N (85.6)
					N (39.7)	**S** (12.7)
					**K** (2.6)	
**D**	MBL-HCV1	4.5	56	48,210	N (78.8)	**S** (78.4)
					**K** (20.2)	N (21.1)
**E**	MBL-HCV1	6.0	28	32,196	**K** (94.2)	N (97.9)
					N (2.7)	
**F**	MBL-HCV1	6.1	42	251,910	N (69.5)	N (61.5)
					**S** (27.1)	**S** (36.5)
					**D** (1.2)	
**G**	Placebo	6.6	7	1,698,789	N (99.1)	N (98.8)
**H**	Placebo	5.5	7	25,752	N (95.9)	N (96.7)
**I**	Placebo	6.0	21	575,572	N (98.6)	N (99.1)
**J**	Placebo	5.1	21*	56	N (99.0)	S (98.7)
**K**	Placebo	5.6	7	2,434,095	N (98.9)	N (99.3)

* - Viral titer <10,000 IU/ml at this time point.

Bold and underlined text represents sequence that diverges from the consensus.

**Figure 1:**
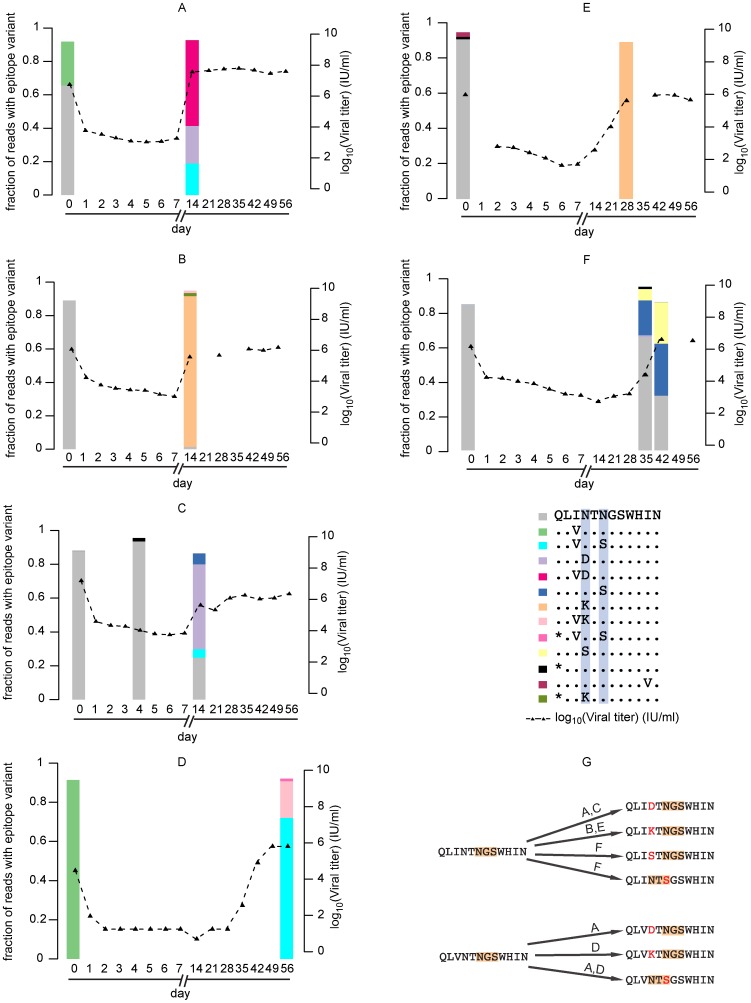
Changes in viral loads and epitope sequence distributions upon MBL-HCV1 antibody administration. **A–F**: Epitope distributions for subjects A, B, C, D, E and F respectively. X axis represents time point in days, 0 denoting pre-transplant baseline sample. Note the depletion of wild-type sequence in all patients and the emergence of resistant epitope sequences (see proportions in left Y axis), in conjunction with viral rebound (right Y axis). Positions 415 and 417 are highlighted in blue in the legend. **G**: Summary of antibody-resistant mutations in epitope sequences, assuming maximum parsimony. Glycosylation motifs are highlighted in orange.

Three of the MAb-treated subjects (Subjects A, B, and C) experienced viral rebound of at least 2 log_10_ IU/ml by day 14 post-transplantation (**Figure 1A–C**). Subject A displayed a mixture of N415D (78.5%) and N417S (20.5%) as the predominant viral strains at the time of rebound. The rebounding population found in subject B was dominated by the variant N415K (97.1%). Subject C had a greater diversity at positions 415 and 417 than Subjects A or B at day 14, with N415D (56.0%), N415K (2.6%), and N417S (12.7%) mixing with quasispecies possessing wild-type epitope sequence (25.8%).

Three of the MAb-treated subjects experienced viral rebound significantly later than day 14 (**Figure 1D–F**). Subject D demonstrated the greatest suppression of HCV viremia following MBL-HCV1 treatment. This subject's viral load transiently fell below the limit of detection and remained below the lower limit of quantification of the HCV RNA assay through day 28. Viral load rebounded 2 log_10_ IU/ml by day 42; however deep sequencing of this serum sample failed for technical reasons. Deep sequencing of the day 56 sample revealed a predominance of the N417S variant (78.4%) and N415K (20.2%). Surprisingly, these two variants were never detected in the same sequence read, indicating that either variant was sufficient for viral escape. For subject E, a post-rebound viral sample obtained on day 28 following transplantation revealed a predominance of N415K (94.2%). Subject F did not experience a 2 log_10_ IU/ml viral rebound until approximately 42 days post-transplantation and maintained wild-type virus (N415/N417) at 34% at this time point. Resistance-associated mutations N415S (27.1%), N415D (1.2%) and N417S (36.5%) were detected by high-throughput sequencing. A previous ad hoc analysis of the clinical data from the pharmacokinetic analysis showed no correlation between the serum antibody concentration and the time to viral rebound (data not shown).

**Figure 2:**
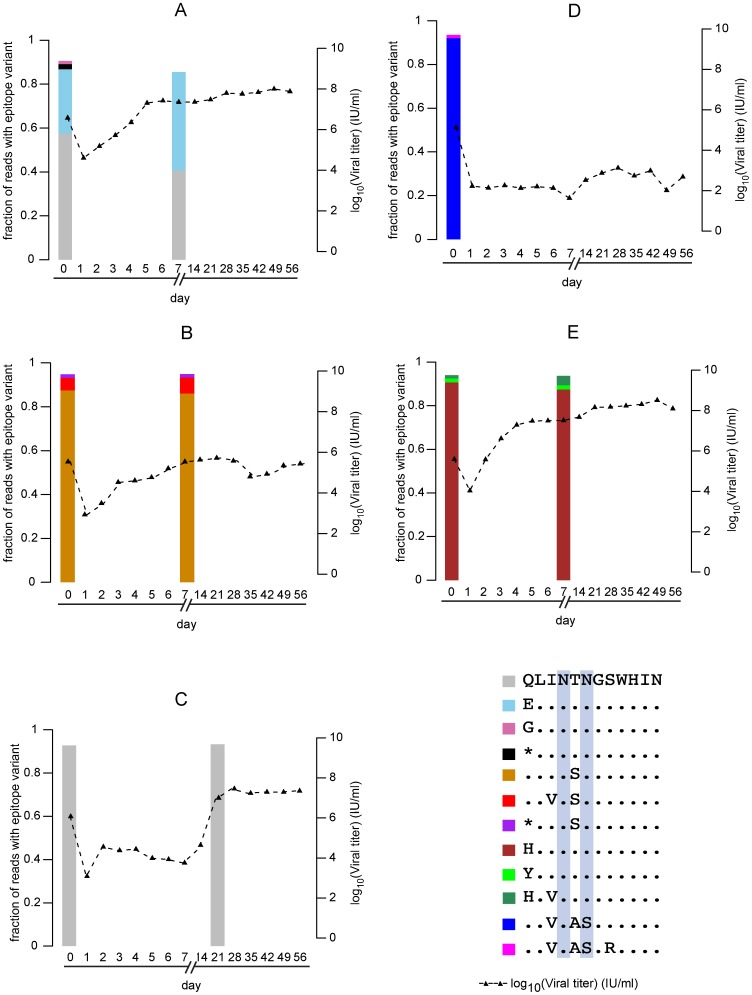
Changes in viral loads and epitope sequence distributions in placebo subjects. **A–E**: Epitope distributions for subjects G, H, I, J and K respectively. X axis represents time point in days, 0 denoting pre-transplant baseline sample. Left Y axis represents proportion of reads with a particular sequence and right Y axis represents viral loads across time points (X axis). Subject J did not have viral titers ≥10,000 IU/ml by day 21; therefore analysis of post-transplant epitope sequence distributions could not be performed.

For four of the five placebo-treated subjects (Subjects G-K), high-throughput sequencing yielded wild type N415/N417 sequences at a frequency of >95% (**Table 1**, **Figure 2**). Interestingly, subject J had a naturally occurring N417S variant that dominated the viral population both pre- and post-transplantation and demonstrated a unique pattern of viral rebound compared to the other placebo subjects. Subject J did not rebound to viral titers ≥10,000 IU/ml post-transplant, limiting the ability to analyze post-transplant sequencing results in this subject. Subject J did not have detectable circulating antibodies to the 412–423 epitope.

### Pre-Transplant Prevalence of Sequence Variation at Amino Acids 415 and 417

With the emergence of four different viral variants following treatment with MBL-HCV1 (N415K, N415D, N415S and N417S), we next determined whether these variants were present before transplantation in any of the 11 subjects. High-throughput sequencing was performed on pre-transplantation serum samples from all subjects. None of the MBL-HCV1-treated subjects (Subjects A–F) had resistance-associated variants (**Figure 1**) present above the detection threshold specified for each sample (**Table S1**) pre-transplantation. Each placebo subject (Subjects G-K) maintained the exact same amino acids at positions 415 and 417 E2 post-transplantation as they had exhibited pre-transplantation (**Table 1**, **Figure 2**), suggesting that these positions are not under selective pressure as the viral quasispecies adapt to the newly transplanted liver.

### Evolution of resistance-associated variants

Since daily serum samples were available for analysis from the first week post-transplantation, high-throughput sequencing was performed on serum samples obtained on Day 4 and Day 7 post-transplantation from the three MBL-HCV1- treated subjects with viral rebound occurring between days 7–14 (Subjects A, B, and C) to explore the evolution of resistance-associated variants. The amount of template in these samples was limited by the low viral load at these early time points (**Figure 1A–C**), ranging from 1,010 IU/ml (Subject B, Day 7) to a maximum of 10,357 IU/ml (Subject C, Day 4). On Day 4 post-transplantation, none of the subjects had detectable levels of variants associated with resistance to MBL-HCV1. By Day 7 post-transplant, neither Subject B nor Subject C had detectable 415 or 417 variants, but Subject A had a dominant population of RAVs (a mixture of N415D and N417S) (data not shown). High-throughput sequencing of additional samples obtained from Subject A during the first post-transplant week revealed detectable resistance-associated variants on Day 3 (N417S, 6.9%), but not on Day 4 or Day 5 (data not shown). By Day 6, however, resistance associated variants (N415D 26.5% and N417S 53.7%) predominated the viral pool, even before the viral load had appreciably increased in this subject).

Given the limited amount of starting template in serum samples from MAb-treated subjects in the first week post-transplant, multiple samples with viral titers <3, 000 IU/ml were sequenced a second time to assess the reproducibility of these results. For subject A when library DNA from the Day 3 sample (1,930 IU/ml), in which low-level RAV (N417S) were detected in the initial analysis, was recreated with the same sets of primers and re-sequenced, the N417S resistance-associated variant was no longer detected. For the Day 4 sample (1,213 IU/ml), E1/E2 DNA sequences were amplified from the original RNA preparation, subjected to 2 additional rounds of re-amplification with subject-specific primers, and sequenced. This technique resulted in the detection of 2 resistance-associated variants that predominated the eventual viral rebound: N415D (1.9%) and N417S (17.4%). The Day 5 sample was extracted from a new aliquot of serum from that time point and amplified using the same techniques as the original Day 5 sample. The repeat of sequencing from this timepoint revealed N415D (13.9%) and N417S (2.8%) resistance-associated variants that were not detected in the original sequencing run. Finally, re-extraction of RNA and re-sequencing of the Day 6 timepoint yielded the same resistance associated variants as the prior run, but at significantly lower frequencies compared with the previous sequencing run (N415D 16.2% vs. 26.5% and N417S 19.0% vs. 53.7%).

These inconsistencies were also observed when low titer samples from a second subject were re-sequenced. For Subject B, the Day 4 sample (2,704 IU/ml) was amplified from the same RNA preparation and analyzed by high throughput sequencing. Interestingly, there were detectable levels of N415K (7.82%), the variant that eventually dominated the viral population at the time of rebound, when this Day 4 sample was re-sequenced. However, when the RNA from the Day 7 sample (1,010 IU/ml) from the same subject (Subject B) was re-extracted, amplified, and re-sequenced, there were no detectable 415 or 417 variants. These findings demonstrate the limitations in the reproducibility of detecting low-level variants contained within low-titer samples.

When serum samples with titers >10,000 IU/ml were subjected to repeat sequencing however, the results were quite reproducible. For example, subject A had a post-rebound viral titer of 35,530,988 IU/ml on Day 14 after transplantation. Two independent amplification and sequencing runs yielded a frequencies of 78.53–83.37% for the N415D variant and 15.54–20.47% for the N417S variant.

### Sequence evolution within the MBL-HCV1 epitope

Having analyzed positions 415 and 417, we also interrogated the entire MBL-HCV1 epitope (amino acids 412–423) to assess whether there were combinations of positions that likely contribute to resistance. We analyzed the sequencing reads that covered the entire epitope and identified the most abundant epitope sequences in each sample. The frequency of each epitope sequence above the detection limit is illustrated in **Figure 1**.

Consistent with the high conservation of this epitope, a majority of reads translated to the sequence QLINTNGSWHIN on day 0 pre-transplantation in four of six MAb-treated subjects (89.0%, 87.8%, 90.8% and 84.4% for patients B, C, E and F, respectively). Two of the MAb-treated subjects had a high frequency of the I414V variant of this canonical sequence (26.2% in subject A and 91.3% in subject D). By the day of viral rebound, the pre-transplant dominant epitope sequence dramatically declined in all MAb-treated subjects. For four MAb-treated subjects (A, B, D, and E), the epitope sequence that dominated pre-transplant was below the detection threshold on the day of rebound. For subjects C and F, the prevalence of the wild-type sequence dropped to 24.8% and 34.6% respectively.

The MAb-resistant epitope sequences contained only point mutations at positions 415 or 417 with respect to the dominant sequences pre-transplantation and all of the resistant variants were obtained by mutating a single nucleotide. The most abundant MAb-resistant variant differed among the subjects (I414V; N415D for subject A, N415K for subjects B and E, N415D for subject C, I414V; N417S for subject D, and N417S for subject F; **Figure 1**). Nonetheless, a glycosylation site is maintained in the variant, with the “N-X-S” motif starting at either positions 415 or 417 (**Figure 1G**). A mutation at position 415 (from unglycosylated N to D, K, S, or glycosylated N) appears required for evasion of MBL-HCV1 neutralization. In comparison, none of these MAb-resistant epitope sequences emerged post-transplant in the subjects treated with placebo (**Figure 2**).

### Sequence evolution outside the MBL-HCV1 epitope

We then expanded our analysis outside the MBL-HCV1 epitope by scanning the entire length of the deeply-sequenced E1/E2 regions to identify other amino acid positions that may be subject to selective pressure in MAb-treated subjects compared to placebo treated subjects. For each position, we performed a chi-square test to quantify the change in the amino-acid distributions between day 0 and the day of rebound, using the day 0 distribution as the expected frequencies and the distribution at rebound as the observed frequencies. A larger chi-square value would therefore correspond to a greater deviation from the day 0 distribution.

**Figure 3:**
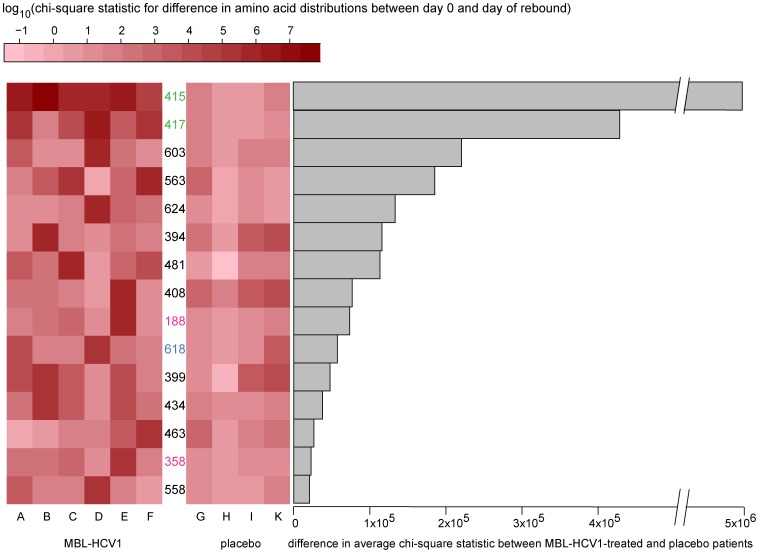
Positions in E1/E2 sequence that show the most significant changes in response to antibody treatment. **Left**: Median-shifted chi-square statistic values of the top 15 amino acid positions across E1 and E2 that show the most difference in average chi-square statistic between MBL-HCV1- and placebo-treated subjects. Placebo subject J was unable to be analyzed due to post-rebound viral titers <10,000 IU/ml. The chi-square statistic quantifies changes in amino acid distributions between day 0 and day of rebound for each subject in each position across the genome. A higher statistic represents a larger change in the amino acid distribution (darker red in heatmap). Positions labeled in green are those within the MBL-HCV1 epitope. Positions labeled in blue are known to participate in CD81 binding. Positions in pink belong to the E1 sequence. **Right**: Differences in mean chi-square statistic between MBL-HCV1-treated and placebo patients. Note that change in position 415 is most significant being 10-fold higher than the second-ranked position 417.

We performed this analysis on all 621 sequenced positions across 6 MBL-HCV1-treated and 4 placebo-treated subjects; subject J in the placebo group could not be analyzed due to low post-rebound viral titers (<10,000 IU/ml). The degree of variation at each amino acid position post-viral rebound was ranked by comparing the difference in the average chi-square values among the MAb-treated subjects and the average chi-square values among the placebo-treated subjects. The heatmap in **Figure 3** shows the normalized variations (absolute difference between each position's chi-square statistic value and the median chi-square value across all positions for the patient) for the 15 most significant amino acid positions. Owing to very high coverage and the sensitivity of this method to coverage, we found that many positions changed significantly between day 0 and the day of rebound. However, the difference in average magnitudes of the amino acid variation between MAb- and placebo-treated subjects at position 415 was 10 times higher than the next highest position, 417 (**Figure 3**), which highlights the importance of position 415 in antibody evasion. Moreover, the heatmap shows that positions 415 and 417 are highly significant across all six MAb-treated subjects and the other positions were only highly significant among subsets of patients. Changes at these other positions co-occur with alterations at position 417 in a subset of MAb-treated subjects (A and D) and could either interfere with MBL-HCV1 binding through an indirect or allosteric effect or could potentially represent compensatory changes which enhance infectivity of the RAVs. Given that the N417S mutation confers full resistance to MBL-HCV1 neutralization [7], it is unlikely that mutations outside of the MBL-HCV1 epitope in conjunction with N417S provide additional resistance to neutralization. These changes may enhance fitness of the N417S variant or they may be fortuitous.

The recently published crystal structure of the HCV E2 core [9] permitted us to examine these positions in greater detail with respect to the MBL-HCV1 epitope and the tertiary structure of E2 (**Table S2**). All of the positions were relatively distant (>18 Å) from residues 421–423 of the epitope (residues 412–420 were disordered in the E2 core structure), and on the opposite side of the E2 core, confirming as noted above that impact on MAb binding, if any, would not be via direct interference. Though they have varying degrees of surface exposure in the E2 core structure, the relatively conservative nature of the amino acid changes before and after MAb treatment, as well as the lack of E1 and remainder of the E2 protein in the structure, makes their functional importance in the context of MAb resistance difficult to assess without additional data.

## Discussion

Treatment at the time of liver transplantation presents a potential opportunity for clearance of hepatitis C infection, given that the primary source of infectious virions is the patient's infected liver [3]. Nearly all HCV-infected patients experience a substantial drop in circulating viral load at the time the diseased liver is removed, only to have viral titers increase rapidly as the donor liver becomes productively infected [2], [3]. An immunoprophylactic strategy to neutralize circulating virions with HCV-specific antibody therapy could exploit this opportunity and protect the allograft in the peri-transplantation period. Human monoclonal antibody MBL-HCV1 provided potent suppression and selective pressure on HCV during the early post-transplant period in a randomized, placebo-controlled trial [8]. Viral load was suppressed in 6 of 6 MAb-treated subjects for time periods ranging from 7 to 28 days, though the virus eventually rebounded with a population dominated by variants with alterations at positions 415 or 417 of the E2 envelope glycoprotein determined by Sanger sequencing [8]. Alterations at these positions have previously been shown to be the main route(s) of escape from MBL-HCV1 neutralization in vitro [6] as well as in vivo in chimpanzees [7].

We developed a high-throughput sequencing methodology to improve the sensitivity to detect low frequency viral strains and further investigate the presence of MAb resistance-associated variants before transplant as well as the development of MAb-escape variants in the peri-transplant and early post-transplant period. Evaluation of rebounding virus load by high-throughput sequencing in MAb-treated subjects identified the same population of escape variants as seen by Sanger sequencing for 5 of the 6 MAb-treated subjects [8]. For Subject C, only high-throughput sequencing detected the N417S variant at 12.7% on day 14, the earliest day rebound was detected. This variant later dominated the viral population when a sample from Day 56 was subject to Sanger sequencing.

High-throughput sequencing was also used to evaluate the pre-transplant viral population in the study subjects. Sanger sequencing of pre-treatment serum samples from subjects in the MAb cohort did not detect any of the resistance-associated variants present at the time of viral rebound [8]. There was a single subject in the placebo group (Subject J) whose dominant viral species was an N417S epitope variant, both pre- and post-transplant. With a sensitivity threshold of 0.5–3.5%, we were unable to detect any additional MAb-resistance associated variants before transplantation. In studies of drugs inhibiting the HCV NS3 protease, the frequency of patients with pre-existing NS3 resistance associated variants was 12% by deep sequencing (0.25% assay cutoff) and 3.5% by Sanger sequencing (20% cutoff) [10], [11]. However, when treated with telaprevir in combination with interferon-α and ribavirin, individuals with resistance-associated variants detectable at baseline by Sanger sequencing had similar rates of sustained virologic response as individuals without detectable baseline resistance-associated variants [10]. As resistance-associated variants to MBL-HCV1 were not detectable in MAb-treated subjects prior to therapy, we are unable to evaluate the virologic response in subjects with pre-existing variation in E1/E2 that could potentially impact neutralization. Given the time required for three of the six subjects to present with viral rebound (>14 days), either RAV were not present in the viral population at the time of treatment or any RAV present prior to treatment required additional mutations to acquire replication fitness in at a minimum these three treated subjects. Given the rapid replication rate of the Hepatitis C virus within in the host, it is unlikely that the differences in time to viral rebound observed within the MAb cohort were a result of varying levels of pre-existing resistance-associated variants prior to transplantation.

We attempted to evaluate the evolution of the viral population in the early post-transplant period; however, our evaluations were severely limited by the low HCV viral load in these samples. This effect was most pronounced in the MAb-treated group, where serum samples with HCV RNA concentrations <1000 IU/ml were unable to be successfully amplified. In addition, the biologic reproducibility of sequencing results from samples containing HCV RNA serum titers between 1000–10,000 IU/ml was poor. Although deep sequencing significantly increases the sensitivity and resolution of sequencing in clinical samples, the ability to draw conclusions from samples with low viral concentrations remains limited by the starting amount of viral template. In low titer samples, each viral template constitutes a larger percentage of the total sample and small variations in amplification efficiency can yield marked changes in final frequency [12].

All of the MBL-HCV1-treated subjects had an alteration at amino acid positions 415 (N415D, N415K, N415S) or 417 (N417S) of the E2 glycoprotein at the time of post-transplant viral rebound. Sequence analysis of the 412–423 epitope in its entirety revealed that the 415 and 417 changes were never observed in the same virus. Interestingly, the N417S mutation mostly likely affected neutralization through its impact on the 415 position, by shifting the N-X-S glycosylation site to the asparagine at position 415. Recent in vitro studies have also confirmed this in vivo observation. Pantua et al. [13] synthesized the N417S variant of E2, observed decreased ability to neutralize virus in vitro with epitope I-directed antibodies, and confirmed glycosylation of the 415 asparagine by mass spectroscopy. The change at position 415 also appeared to be sufficient to confer resistance, in an otherwise replication competent virus, as there were no other sites on the E1/E2 glycoprotein that were consistently mutated at the time of rebound in multiple subjects. This is supported by the exposed “flap-like” structure of the MBL-HCV1 epitope [14] that is apparently not embedded in the E2 core structure [9], reducing the likelihood of interaction with other residues at least within the main core structure (positions 412–645).

There are both pre-clinical as well as emerging clinical evidence to suggest that addition of a second antiviral agent with a discrete mechanism of action can impair the development of resistance. The addition of the licensed HCV NS3 protease inhibitor telaprevir to an antibody binding the E2 412–423 epitope in vitro led to a synergistic negative effect on viral replication and suppressed the emergence of resistance to both agents [13]. Given that the baseline frequency of HCV variants with resistance associated mutations in E2 412–423 appears to be low and that post-transplant viral rebound is delayed until after day 7, antibody-based peri-transplant treatment could provide a safe, well-tolerated therapy that can be started at the time of transplant surgery and allow a window of a few days for a small molecule antiviral agent targeting a different stage of the viral life cycle to be added once the patient has stabilized and graft function is established. Such combination therapy holds the potential to eliminate the hepatitis C virus before a chronic infection can be established in the newly transplanted liver.

## Materials and Methods

### Ethics Statement

The clinical trial was registered at ClinicalTrials.gov (Registration number NCT01121185). Details of the trial are also detailed in Chung et al [8]. The protocol was approved by each study site's institutional review board and conducted in accordance with Good Clinical Practice guidelines set by the International Conference on Harmonization. All subjects provided written informed consent. The following is the list of IRBs of the study sites that conducted the clinical trials.

- Yale-New Haven Hospital IRB, New Haven, Connecticut, United States, 06504
- Beth Israel Deaconess Medical Center IRB, Boston, Massachusetts, United States, 02215
- Massachusetts General Hospital IRB, Boston, Massachusetts, United States, 02114
- Lahey Clinic IRB, Burlington, Massachusetts, United States, 01805
- Henry Ford Health System IRB, Detroit, Michigan, United States, 48202
- Mount Sinai Hospital IRB, New York, New York, United States, 10029
- Cleveland Clinic Foundation IRB, Cleveland, Ohio, United States, 44195
- Methodist Healthcare Foundation IRB, Memphis, Tennessee, United States, 38104

### Collection of Serum Samples for Viral Sequence Analyses

Serum samples for HCV sequence analyses were obtained at protocol-specified timepoints as part of a randomized, double-blind, placebo-controlled trial of MBL-HCV1 treatment in liver transplant patients [8]. Eleven subjects with HCV genotype 1a infection were enrolled; six subjects received 11 study infusions of MBL-HCV1 (50 mg/kg each) and five subjects received 11 infusions of placebo (0.9% sodium chloride, starting right before transplant surgery and continuing for 14 days. Serum samples for assessment of HCV RNA titers and for viral sequence analyses were obtained prior to transplant, daily for the first post-transplant week, and weekly through day 56±2 post-transplantation. Serum HCV RNA levels were measured at ICON Central Laboratories (Farmingdale, NY) using the COBAS Ampliprep/COBAS TaqMan HCV Test (Roche Molecular Diagnostics) as previously described [8].

### E1/E2 Amplification and High-throughput Sequencing

Viral RNA was extracted from 1 ml of human sera using the QIAamp viral RNA mini kit (Qiagen) as described by the manufacturer to a final volume of 240 microliters. Three microliters of viral RNA in six independent reactions for each sample was amplified using the Superscript III One-Step RT-PCR system with Platinum Taq (Invitrogen). For specific amplification, a forward oligonucleotide containing a HindIII restriction site (5′-gct tag caa gct tCG CCG ACC TCA TGG GGT ACA TAC CGC TCG-3′) targeting the gene encoding core, upstream of the E1 gene and a reverse oligonucleotide containing an XbaI restriction site (5′-cgc ttg ctc tag aCG AGG TT CTC CAA AGC CGC CTC CGC TTG G-3′) annealing to the 3′ end of E2 were designed. RNA was reverse transcribed and DNA amplified for 30–40 cycles. RT-PCR resulted in an amplicon approximately 1890 bp in size representing the entire E1/E2 coding region.

For serum samples obtained at baseline or following 2 log_10_ viral rebound, the six independent reactions for each serum sample were pooled, resolved on agarose gels and gel purified. A portion of the sample was cloned into pcDNA3.1 using HindIII/XbaI and conventional Sanger sequencing was performed on 8–20 unique clones. Approximately 1–5 micrograms of RT-PCR product was sheared using a Covaris S-series SonoLab Single sonicator to obtain DNA fragments near 225 bp in size. The 5′ and 3′ ends of the sheared DNA were repaired and blunted using the End-It DNA End-Repair Kit (Epicentre). Blunted DNA was A-tailed using the Exo-minus Klenow kit (Epicentre). Illumina paired-end adapters containing 5-mer barcodes were ligated to the A-tailed DNA and the reaction products were resolved on 2% agarose gels. Adaptered material in the range of 200–250 bp was gel purified and amplified (18 cycles) with Platinum Pfx (Invitrogen) and Illumina paired-end primers 1.01 and 2.01. Amplified material was purified using a QiaQuick PCR clean up kit (Qiagen). Purified material was assessed for concentration and library size distribution using an Agilent 2100 Bioanalyzer G2939A with the Agilent Bioanalyzer High Sensitivity DNA Kit.

For serum samples obtained prior to 2 log_10_ post-transplant viral rebound, a nested PCR was employed using subject-specific primers designed based on Sanger sequencing results of pre-transplant and post-rebound timepoints. Briefly, sequences upstream of the E1 gene were analyzed for each individual subject and a region with no nucleotide variability (pre-transplant or post-viral rebound) was selected for primer design. The reverse primer was the same as used in the One-Step RT-PCR and the forward primers were as follows: Subject A, 5′-gct tag caa gct tGC TGC CAG GGC CCT GGC GCA TGG C-3′; Subject B, 5′-gct tag caa gct tGG CGT CCG GGT TCT GGA AGA CGG CGT G-3′; Subject C, 5′-gct tag caa gct tCC CTG GCG CAC GGC GTC CGG GTT C-3′, Subject F, 5′-gct tag caa gct tCT GGC GCA TGG CGT CCG GGT CCT GG-3′. Reamplified products were prepared for high-throughput sequencing as described above.

### High-Throughput Sequence Acquisition and Data Analysis

All samples were sequenced using an Illumina HiSeq 2000 instrument to yield 100-nucleotide paired-end reads. **Table S3** provides detailed statistics on the sequencing datasets of all samples. Sequence quality was assessed using the FastQC software {http://www.bioinformatics.babraham.ac.uk/projects/fastqc/} and samples with inadequate quality were re-sequenced. In order to maintain comparable sequencing accuracies between samples, only reads with a quality score of 28 or higher for the first 50 bases were included in the analyses. These reads were mapped to a database of 581 E1/E2 HCV1 sequences obtained by Sanger sequencing from 27 HCV-infected individuals, including the 11 subjects in the current protocol. The BWA software [15] was used for read mapping, allowing a maximum of 5 mismatches per read. Datasets with an average fold-coverage of less than 40,000 reads per base were discarded. If multiple sequencing runs of the same sample passed the above criterion, the dataset with the greatest fold coverage was used for downstream analysis.

Sequencing error for each dataset was estimated by computing the mean and standard deviation of error per base (percentage of reads with incorrect base calls) in the invariant 3′-end primer region. The detection threshold for each sample was defined as the mean plus one standard deviation of the error rate, i.e., a nucleotide variant is considered to be present in a sample if its frequency is higher than the detection threshold. The sensitivity thresholds ranged from 0.5–3.5% across the datasets (**Table S1**).

## Supporting Information

Table S1 **Base accuracy calculated from the 3′ end primer sequences.**
(DOCX)

Table S2 **Significant amino acid changes outside of the MBL-HCV1 epitope and their physical distance from the MBL-HCV1 epitope based on the E2 core structure.**
(DOCX)

Table S3 **Sequencing Statistics of all deep-sequenced samples.**
(DOCX)

Protocol S1 **Clinical Trial Protocol.**
(DOCX)
