# Supplementary material for: High-Throughput Sequencing Analysis of Post-Liver Transplantation HCV E2 Glycoprotein Evolution in the Presence and Absence of Neutralizing Monoclonal Antibody
Source: PLoS One. 2014 Jun 23;9(6):e100325. doi: 10.1371/journal.pone.0100325 (PMC4067308; doi:10.1371/journal.pone.0100325)
Supplement: Table S1 — Base accuracy calculated from the 3′ end primer sequences. (DOCX) [file pone.0100325.s001.docx]

**Table S1. Base accuracy calculated from the 3´ end primer sequences.**

| **Subject** | **Treatment** | **Study Day** | **Mean Accuracy Across Primer Positions (%)** | **Standard Deviation (S.D.) of Accuracy (%)** | **Sequencing Error Cutoff (Mean Error + 1 S.D., %)** |
| --- | --- | --- | --- | --- | --- |
| A | MBL-HCV1 | 0 | 98.65 | 1.53 | 2.88 |
|  |  | 1 | 99.72 | 0.40 | 0.68 |
|  |  | 2 | 99.75 | 0.26 | 0.52 |
|  |  | 3 | 99.76 | 0.23 | 0.47 |
|  |  | 4 | 99.63 | 0.42 | 0.79 |
|  |  | 5 | 99.70 | 0.23 | 0.54 |
|  |  | 6 | 99.69 | 0.40 | 0.71 |
|  |  | 7 | 99.65 | 0.31 | 0.66 |
|  |  | 14 | 99.32 | 0.69 | 1.36 |
| B | MBL-HCV1 | 0 | 99.02 | 1.19 | 2.17 |
|  |  | 4 | 99.72 | 0.31 | 0.59 |
|  |  | 7 | 99.4 | 0.92 | 1.44 |
|  |  | 14 | 99.69 | 0.44 | 0.75 |
| C | MBL-HCV1 | 0 | 99.18 | 1.37 | 2.20 |
|  |  | 4 | 99.67 | 0.45 | 0.78 |
|  |  | 7 | 99.52 | 0.66 | 1.14 |
|  |  | 14 | 99.24 | 1.63 | 2.39 |
| D | MBL-HCV1 | 0 | 99.44 | 0.80 | 1.36 |
|  |  | 56 | 99.56 | 0.56 | 0.99 |
| E | MBL-HCV1 | 0 | 99.69 | 0.50 | 0.81 |
|  |  | 28 | 99.19 | 1.73 | 2.54 |
| F | MBL-HCV1 | 0 | 98.93 | 2.38 | 3.45 |
|  |  | 4 | 99.75 | 0.34 | 0.59 |
|  |  | 28 | 99.73 | 0.31 | 0.58 |
|  |  | 35 | 99.74 | 0.35 | 0.61 |
|  |  | 42 | 99.20 | 1.36 | 2.16 |
| G | Placebo | 0 | 99.62 | 0.72 | 1.10 |
|  |  | 7 | 99.51 | 0.87 | 1.36 |
| H | Placebo | 0 | 99.71 | 0.36 | 0.64 |
|  |  | 7 | 99.62 | 0.59 | 0.97 |
| I | Placebo | 0 | 99.38 | 1.36 | 1.98 |
|  |  | 21 | 99.44 | 1.13 | 1.69 |
| J | Placebo | 0 | 99.56 | 0.69 | 1.14 |
|  |  | 21 | 99.58 | 0.58 | 1.00 |
| K | Placebo | 0 | 99.60 | 0.70 | 1.09 |
|  |  | 7 | 99.53 | 0.76 | 1.23 |
