# Supplementary material for: High-Throughput Sequencing Analysis of Post-Liver Transplantation HCV E2 Glycoprotein Evolution in the Presence and Absence of Neutralizing Monoclonal Antibody
Source: PLoS One. 2014 Jun 23;9(6):e100325. doi: 10.1371/journal.pone.0100325 (PMC4067308; doi:10.1371/journal.pone.0100325)
Supplement: Table S2 — Significant amino acid changes outside of the MBL-HCV1 epitope and their physical distance from the MBL-HCV1 epitope based on the E2 core structure. (DOCX) [file pone.0100325.s002.docx]

**Table S2. Significant amino acid changes outside of the MBL-HCV1 epitope and their physical distance from the MBL-HCV1 epitope based on the E2 core structure.**

| **Position** | **Amino acid** | **Side chain % ASA** | **Distance from MBL-HCV1 epitope (Å)** | **Amino acid change** |
| --- | --- | --- | --- | --- |
| 603 | I | 2.1 | 23.8 | I  L |
| 563 | V | 31.1 | 21.0 | V  A |
| 624 | Y | 20.6 | 26.2 | Y  F |
| 618 | Y | 7.4 | 20.2 | Y  F |
| 558 | T | 61.3 | 18.2 | T  S |
| 434 | N | 77.1 | 23.9 | K🡺 N |
