# Supplementary material for: High-Throughput Sequencing Analysis of Post-Liver Transplantation HCV E2 Glycoprotein Evolution in the Presence and Absence of Neutralizing Monoclonal Antibody
Source: PLoS One. 2014 Jun 23;9(6):e100325. doi: 10.1371/journal.pone.0100325 (PMC4067308; doi:10.1371/journal.pone.0100325)
Supplement: Table S3 — Sequencing Statistics of all deep-sequenced samples. (DOCX) [file pone.0100325.s003.docx]

**Table S3: Sequencing statistics of all deep-sequenced samples**

| Subject | Study day | Treatment | Viral titer  (IU/ml) | Total reads | % reads mapped to reference | Mean  fold  coverage  (reads per base) |
| --- | --- | --- | --- | --- | --- | --- |
| A | 0 | MBL-HCV1 | 5,496,127 | 14,309,904 | 41.92 | 301,518 |
|  | 1 |  | 5,822 | 6,450,444 | 79.71 | 260,696 |
|  | 2 |  | 3,296 | 23,924,710 | 80.08 | 972,741 |
|  | 3 |  | 1,930 | 30,334,822 | 78.89 | 1,215,659 |
|  | 4 |  | 1,213 | 16,996,320 | 64.63 | 551,258 |
|  | 5 |  | 1,065 | 22,760,974 | 80.28 | 925,901 |
|  | 6 |  | 1,163 | 28,112,544 | 70.69 | 1,011,267 |
|  | 7 |  | 1,820 | 6,104,432 | 66.62 | 205,630 |
|  | 14 |  | 35,530,988 | 9,765,532 | 47.41 | 234,795 |
| B | 0 | MBL-HCV1 | 1,135,133 | 16,031,764 | 37.53 | 302,612 |
|  | 4 |  | 2,704 | 20,986,398 | 68.63 | 721,150 |
|  | 7 |  | 1,010 | 4,066,710 | 62.67 | 128,978 |
|  | 14 |  | 362,858 | 32,504,392 | 68.74 | 1,133,834 |
| C | 0 | MBL-HCV1 | 15,423,018 | 2,786,702 | 33.45 | 46,934 |
|  | 4 |  | 10,357 | 21,453,138 | 55.81 | 605,487 |
|  | 7 |  | 6,880 | 415,558 | 70.13 | 14,771 |
|  | 14 |  | 427,166 | 11,086,950 | 48.58 | 272,471 |
| D | 0 | MBL-HCV1 | 29,616 | 12,264,006 | 49.68 | 310,338 |
|  | 56 |  | 642,797 | 11,153,416 | 47.76 | 270,719 |
| E | 0 | MBL-HCV1 | 989,109 | 10,618,328 | 50.60 | 273,647 |
|  | 28 |  | 429,279 | 6,595,232 | 27.15 | 91,218 |
| F | 0 | MBL-HCV1 | 1,207,945 | 5,094,372 | 35.86 | 93,063 |
|  | 4 |  | 5,875 | 17,530,674 | 53.24 | 474,167 |
|  | 28 |  | 1,342 | 27,525,394 | 71.79 | 1,006,162 |
|  | 35 |  | 20,657 | 19,969,004 | 74.12 | 753,599 |
|  | 42 |  | 3,358,802 | 4,143,238 | 32.85 | 69,326 |
| G | 0 | Placebo | 3,786,170 | 22,774,650 | 52.75 | 611,973 |
|  | 7 |  | 22,650,518 | 11,865,958 | 58.16 | 350,904 |
| H | 0 | Placebo | 342,731 | 22,030,844 | 66.79 | 746,145 |
|  | 7 |  | 343,362 | 22,710,172 | 58.73 | 677,817 |
| I | 0 | Placebo | 1,083,060 | 26,523,166 | 51.69 | 690,344 |
|  | 21 |  | 7,674,293 | 21,575,812 | 51.68 | 566,732 |
| J | 0 | Placebo | 132,685 | 20,094,210 | 50.40 | 515,376 |
|  | 21 |  | 746 | 19,675,784 | 55.66 | 557,882 |
| K | 0 | Placebo | 386,586 | 23,713,448 | 44.32 | 535,380 |
|  | 7 |  | 32,454,598 | 17,013,526 | 28.21 | 244,203 |
