# Supplementary material for: High-Throughput Sequencing Analysis of Post-Liver Transplantation HCV E2 Glycoprotein Evolution in the Presence and Absence of Neutralizing Monoclonal Antibody
Source: PLoS One. 2014 Jun 23;9(6):e100325. doi: 10.1371/journal.pone.0100325 (PMC4067308; doi:10.1371/journal.pone.0100325)
Supplement: Protocol S1 — Clinical Trial Protocol. (DOCX) [file pone.0100325.s004.docx]

**Protocol S1: Clinical Trial Protocol (as detailed in** [**www.clinicaltrials.gov**](http://www.clinicaltrials.gov)**, identifier number NCT01121185)**

**Title of study**

A Phase II Randomized, Double-Blind, Placebo Controlled Study of the Clinical Effectiveness of a Human Monoclonal Antibody Against Hepatitis C Virus E2 Glycoprotein (MBL-HCV1) in Hepatitis C Infected Patients Undergoing Liver Transplantation

**Brief Summary**

The purpose of this study is to determine whether a human monoclonal antibody against Hepatitis C (MBL-HCV1) is effective in preventing detectable levels of Hepatitis C virus in patients undergoing liver transplantation due to chronic HCV infection. The study will also determine if MBL-HCV1 is effective in delaying or reducing the amount of detectable HCV in patients after transplant.

**Detailed Description**

This is a Phase 2, randomized, double-blind, placebo controlled study in Hepatitis C (HCV) infected patients undergoing liver transplantation. Chronically infected patients with HCV genotype 1a scheduled to receive a liver transplant from either a deceased or living donor who satisfy all study inclusion or exclusion criteria will be approached to participate. The study will be conducted in two parts to test a human monoclonal antibody against Hepatitis C (MBL-HCV1). In Part 1, sixteen eligible patients will be randomized 1:1 to receive 50 mg/kg MBL-HCV1 or 0.9% sodium chloride placebo intravenously. Eleven doses will be given during the first 14 days post transplantation. Patients will be evaluated through day 56 for safety and clinical outcomes that include measurement of anti-HCV antibodies, anti-drug antibody and HCV viral load. On study visit day 42, a liver biopsy will be performed for evaluation of hepatitis. Physical examination, vital sign measurements, emergence of adverse events and concomitant medication usage will be assessed at scheduled visits and as needed during the 56 day study period.

The Data Safety and Monitoring Board will perform a futility analysis after the first 16 patients have been enrolled and completed study follow-up through study visit day 42 post transplant. Based on the results of the interim analysis, the dose of MBL-HCV1 for part 2 of the study will be determined. Part 2 of the study will be conducted in the same manner as Part 1.
